# Pathogenic *VCP* Mutations Induce Mitochondrial Uncoupling and Reduced ATP Levels

**DOI:** 10.1016/j.neuron.2013.02.028

**Published:** 2013-04-10

**Authors:** Fernando Bartolome, Hsiu-Chuan Wu, Victoria S. Burchell, Elisavet Preza, Selina Wray, Colin J. Mahoney, Nick C. Fox, Andrea Calvo, Antonio Canosa, Cristina Moglia, Jessica Mandrioli, Adriano Chiò, Richard W. Orrell, Henry Houlden, John Hardy, Andrey Y. Abramov, Helene Plun-Favreau

**Affiliations:** 1 Department of Molecular Neuroscience, UCL Institute of Neurology, Queen Square, London WC1N 3BG, UK; 2 Department of Neurodegenerative Disease, Dementia Research Centre, UCL Institute of Neurology, Queen Square, London WC1N 3BG, UK; 3 Reta Lilla Weston Laboratories, UCL Institute of Neurology, Queen Square, London WC1N 3BG, UK; 4 Department of Clinical Neurosciences, UCL Institute of Neurology, Rowland Hill Street, London NW3 2PF, UK; 5 Department of Neuroscience, University of Turin, 10126 Turin, Italy; 6 Department of Neuroscience, S. Agostino-Estense Hospital and University of Modena, 41126 Modena, Italy

## Abstract

Valosin-containing protein (VCP) is a highly expressed member of the type II AAA+ ATPase family. *VCP* mutations are the cause of inclusion body myopathy, Paget’s disease of the bone, and frontotemporal dementia (IBMPFD) and they account for 1%–2% of familial amyotrophic lateral sclerosis (ALS). Using fibroblasts from patients carrying three independent pathogenic mutations in the *VCP* gene, we show that VCP deficiency causes profound mitochondrial uncoupling leading to decreased mitochondrial membrane potential and increased mitochondrial oxygen consumption. This mitochondrial uncoupling results in a significant reduction of cellular ATP production. Decreased ATP levels in VCP-deficient cells lower their energy capacity, making them more vulnerable to high energy-demanding processes such as ischemia. Our findings propose a mechanism by which pathogenic *VCP* mutations lead to cell death.

## Highlights

► VCP deficiency is associated with mitochondrial depolarization ► VCP deficiency leads to increased mitochondrial respiration and uncoupling ► ATP levels are decreased in VCP-deficient cells due to lower ATP production

## Teaser

In this study, Bartolome et al. show that three independent pathogenic VCP mutations induce mitochondrial uncoupling, resulting in low cellular ATP production, rendering the cells more susceptible to cell death under stress-induced ischemic conditions.

## Introduction

Valosin-containing protein (VCP), also referred to as p97, is a highly expressed member of the type II AAA+ (ATPase associated with multiple activities) ATPase family. Single missense mutations in the *VCP* gene are the cause of frontotemporal dementia (IBMPFD) (Kimonis et al., 2000; Watts et al., 2004) and may account for 1%–2% of familial amyotrophic lateral sclerosis (ALS) (Johnson et al., 2010). However, the molecular mechanisms by which VCP deficiency contributes to these diseases are yet to be determined. ALS and frontotemporal dementia (FTD) are clinically distinct disorders that have recently been brought together with the identification of C9orf72 expansions and the important neuropathological overlap of cytoplasmic inclusions of TAR DNA binding protein 43 (TDP-43) in both disorders.

VCP was shown to play a role in seemingly unrelated cellular processes (for review, see Meyer et al., 2012; Yamanaka et al., 2012). The high homology of *VCP* between species (CDC48 in yeast, TER94 in *Drosophila*, and p97 in mouse) has allowed the design of powerful model organisms aimed at studying the molecular mechanisms associated with VCP deficiency and *VCP* pathogenic mutations (Badadani et al., 2010; Custer et al., 2010; Weihl et al., 2007). In particular, the recently reported ^R155H/+^ *VCP* knockin mice show extensive accumulation of abnormal mitochondria (Nalbandian et al., 2013; Yin et al., 2012). Mitochondria play key roles in numerous cellular processes, including ATP production, calcium homeostasis, and ultimately cell death. In no cell type is their function more vital than in neurons, in which limited glycolysis causes the cell to rely on oxidative phosphorylation for ATP production. In this study, we used dynamic imaging techniques to explore the mitochondrial pathophysiology in a *VCP* knockdown (*VCP* KD) human dopaminergic neuroblastoma cell line (SH-SY5Y) and in fibroblasts from patients carrying three independent pathogenic mutations in the *VCP* gene. We demonstrate that VCP deficiency induces the uncoupling of respiration from oxidative phosphorylation. This results in decreased mitochondrial membrane potential, leading to higher respiration and lower ATP levels due to reduced ATP production. These findings define a mechanism whereby VCP dysfunction may cause cell death and highlight pathophysiological events that may occur in IBMPFD.

## Results

### VCP Deficiency Is Associated with Decreased Mitochondrial Membrane Potential

**Figure 1:**
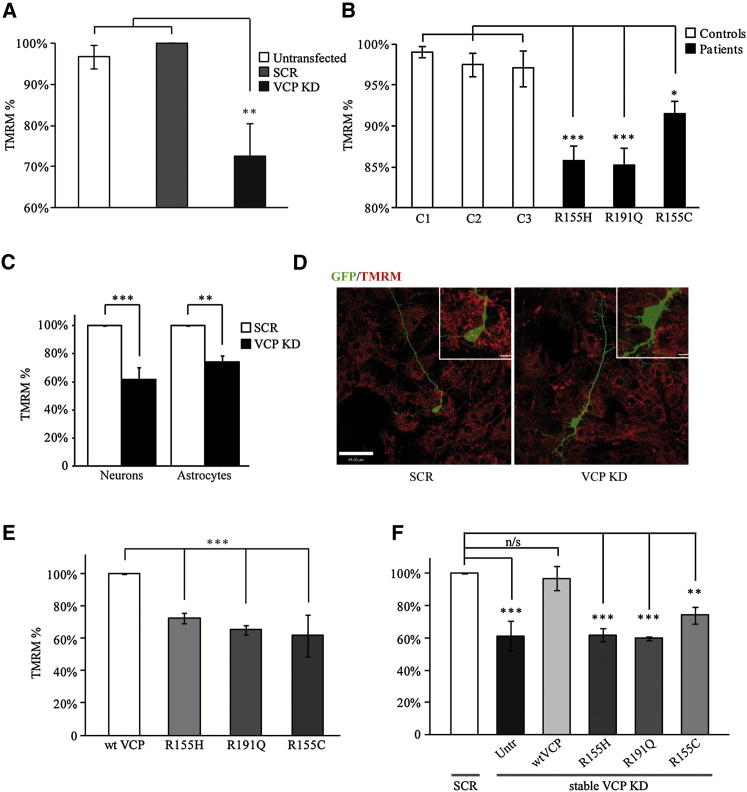
Mitochondria Are Depolarized in VCP-Deficient Cells (A–C) Mitochondrial membrane potential (ΔΨ_m_) was estimated by live cell imaging using TMRM in redistribution mode (40 nM) in different cell models. (A) SH-SY5Ys left untransfected, transfected with scramble (SCR), or *VCP* siRNA. (B) Patient fibroblasts carrying the *VCP* pathogenic mutations R155H, R191Q, and R155C compared to age-matched control fibroblasts (C1, C2, and C3). (C) Mouse cortical primary neurons and astrocytes transiently transduced with SCR or *VCP* shRNA. (D) Images from neurons transduced with GFP SCR shRNA or GFP *VCP* KD shRNA (green, GFP; red, TMRM). Scale bar represents 44 μm; in inset images, scale bars represent 6.3 μm and 5.5 μm for SCR and *VCP* KD, respectively. (E) ΔΨ_m_ was measured in the SH-SY5Y cells overexpressing WT or mutant VCP. (F) ΔΨ_m_ was measured in stable SCR and *VCP* KD SH-SY5Y cells left untransfected, overexpressing WT, or mutant *VCP*. In each experiment, data were normalized to control SCR cells (A, C, and F), control fibroblasts (B), and WT *VCP* overexpressing cells (E) and are represented as the mean ± SEM from at least three independent experiments (^∗^p < 0.05; ^∗∗^p < 0.01; ^∗∗∗^p < 0.005 compared with control cells as calculated by one-way ANOVA). See also Figures S1 and S2.

Mitochondrial membrane potential (ΔΨ_m_) is an indicator of mitochondrial health and function. To study VCP implication in mitochondrial function, we transiently silenced the *VCP* gene using siRNA in SH-SY5Y human neuroblastoma cells (see Figure S1A available online) and shRNA in mouse primary cortical cultures (Figure 1D). Additionally, stable populations of *VCP* KD SH-SY5Y cells were generated using shRNA (Figure S1B). ΔΨ_m_ was measured in VCP-deficient SH-SY5Y cells (Figure 1A), in human fibroblasts from three patients with independent *VCP* mutations (R155C, R155H, and R191Q; for donors' details see Figure S1E and Table S1) and age-matched controls (Figure 1B), and in primary neurons and astrocytes (Figures 1C and 1D). A significant decrease in ΔΨ_m_ was observed in all VCP-deficient cell models studied (SH-SY5Y cells = 72% ± 8%, n > 20 cells in 3 independent experiments compared to either untransfected cells or cells transfected with scramble (SCR) control siRNA; primary neurons = 62% ± 9% and primary astrocytes = 74% ± 4%, n ≥ 5 cells in 3 independent experiments compared to cells transfected with SCR control shRNA; fibroblasts from patient 1 = 86% ± 2%, n = 7; fibroblasts from patient 2 = 85% ± 2%, n = 8; fibroblasts from patient 3 = 91% ± 2%, n = 5, compared to age-matched control fibroblasts) (Figures 1A–1C). Overexpression of R155H, R191Q, and R155C VCP mutants in SH-SY5Y cells is associated with a significant reduction in the TMRM signal (TMRM in cells overexpressing R155H VCP = 73% ± 3%; R191Q VCP = 65% ± 3%; R155C VCP = 62% ± 13% compared to overexpressed WT VCP; n ≥ 3), confirming that the three pathogenic *VCP* mutations have a dominant-negative effect (Figure 1E). Re-expression of WT, but not mutant *VCP*, rescued the TMRM signal in a clonal population of stable *VCP* KD SH-SY5Y cells (untransfected cells = 61% ± 9%; WT *VCP* = 97% ± 7%; R155H *VCP* = 62% ± 4%; R191Q *VCP* = 60% ± 1%; R155C *VCP* = 74% ± 5% compared to SCR SH-SY5Y control cells; n ≥ 3) (Figures 1F and S1D).

In healthy cells, ΔΨ_m_ is maintained by mitochondrial respiration. Upon respiratory dysfunction, cells may maintain ΔΨ_m_ using ATP hydrolysis by the ATP synthase (reviewed in Nicholls, 2002). In order to investigate the mechanism of maintenance of ΔΨ_m_, a series of mitochondrial toxins were applied and their effects on ΔΨ_m_ were observed. All control cells and *VCP* KD SH-SY5Y cells showed no significant response to the F_1_F_0_-ATP synthase inhibitor oligomycin (0.2 μg/ml), while subsequent inhibition of complex I by rotenone (5 μM) caused a rapid loss of potential (Figure S2A). However, application of oligomycin to patient fibroblasts carrying *VCP* mutations resulted in a modest depolarization, suggesting that complex V may be partially working in reverse mode in these cells, in order to maintain the ΔΨ_m_ (Figure S2B). Application of rotenone (5 μM) to inhibit complex I then generated a strong depolarization. Complete depolarization was assessed in all cell models by addition of the mitochondrial uncoupler carbonylcyanide-p-trifluoromethoxyphenylhydrazone (FCCP) (1 μM) (Figure S2B). Taken together, these data suggest that ΔΨ_m_ is mainly maintained by respiration in VCP-deficient cells.

### Mitochondrial Respiration Is Increased in VCP-Deficient Cells

**Figure 2:**
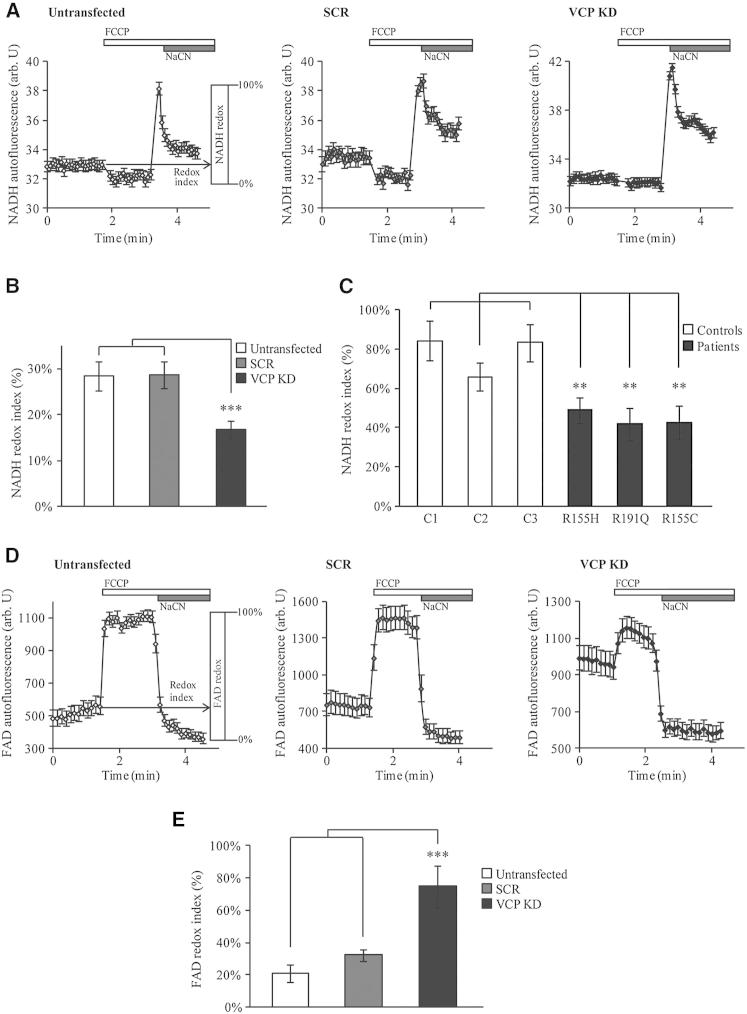
Mitochondrial Respiration Is Increased in VCP-Deficient Cells (A–C) NADH redox indexes were obtained after monitoring NADH autofluorescence in VCP-deficient cells compared to control cells. (A) Representative experiments of NADH autofluorescence from untransfected, SCR, and *VCP* KD cells (mean ± SEM of at least 20 cells on a single coverslip) in which the NADH redox index (the initial redox level expressed as percentage of the range) is described graphically (left). NADH redox indexes were obtained by calculating the initial NADH autofluorescence when the minimum NADH autofluorescence after FCCP (1 μM) addition is normalized to 0% and the maximum to 100% after addition of NaCN (1 mM). (B) NADH redox indexes from untransfected, SCR, and *VCP* KD SH-SY5Y cells. (C) NADH redox indexes from control and VCP-deficient fibroblasts. (D and E) FAD autofluorescence was monitored by confocal microscopy in untransfected, SCR, and *VCP* KD SH-SY5Y cells. (D) Representative experiments of FAD autofluorescence from untransfected, SCR, and *VCP* KD cells (mean ± SEM of at least 20 cells on a single coverslip) in which the FAD/redox index (the initial redox level expressed as percentage of the range) is described graphically (left). FAD redox indexes were obtained by calculating the initial FAD autofluorescence after normalizing the FCCP (1 μM) response to 100% (maximum respiration) and the NaCN (1 mM) response to 0% (minimum respiration). (E) FAD redox indexes from untransfected, SCR, and *VCP* KD SH-SY5Y cells. In all cases, error bars represent the mean ± SEM of at least three independent experiments (^∗∗^p < 0.01; ^∗∗∗^p < 0.005, compared with control cells as calculated by one-way ANOVA).

The redox state of NADH or FAD reflects the activity of the mitochondrial electron transport chain (ETC) and the rate of substrate supply. We measured the basal levels of NADH (substrate for the ETC complex I) and FAD autofluorescence and generated the “redox indexes” by expressing basal NADH or FAD levels as a percentage of the difference between the maximally oxidized and maximally reduced signals. The maximally oxidized signal is defined as the response to 1 μM FCCP that stimulates maximal respiration, while the maximally reduced signal is defined as the response to 1 mM NaCN that fully inhibits respiration. Figure 2A shows average traces for NADH autofluorescence in untransfected, SCR, and *VCP* KD SH-SY5Y cells. The NADH redox index generated was significantly lower in transient *VCP* KD SH-SY5Y cells (17% ± 2%, n = 8) compared to control untransfected (28% ± 3%, n = 8) and SCR-transfected (29% ± 3%, n = 8) cells (Figure 2B), indicating a depletion of NADH under basal conditions. NADH redox index in patient fibroblasts was also lower than in the age-matched controls (patient 1 = 49% ± 7%, n = 9; patient 2 = 48% ± 8%, n = 8; patient 3 = 43% ± 9%, n = 10; control 1 = 84% ± 10%, n = 7; control 2 = 66% ± 7%, n = 7; control 3 = 83% ± 9%, n = 8) (Figure 2C).

We then measured the FAD autofluorescence in SH-SY5Y cells. Figure 2D shows average traces for FAD in untransfected, SCR, and *VCP* KD SH-SY5Y cells. The generated FAD redox index was significantly higher in transient *VCP* KD SH-SY5Y cells (75% ± 13%, n = 4) compared to control untransfected (21% ± 5%; n = 4) and SCR-transfected (32% ± 4%; n = 4) cells (Figure 2E). We were unable to measure the FAD redox state in fibroblasts due to the very low level of FAD autofluorescence in these cells.

### Oxygen Consumption Is Increased in VCP-Deficient Cells

**Figure 3:**
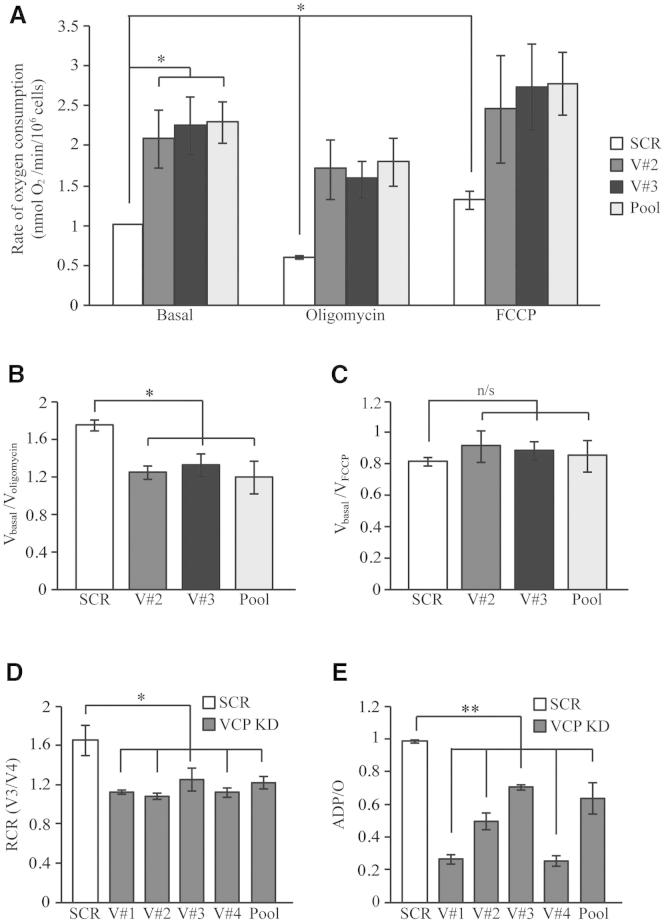
Oxygen Consumption Is Increased in VCP-Deficient Cells (A–C) Oxygen consumption was measured in intact SH-SY5Y cells using a Clark oxygen electrode. (A) Basal respiration (V_basal_) was measured in stable SCR and *VCP* KD cells (clones V#2, V#3, and Pool). ATP production was then inhibited using oligomycin (0.2 μg/ml) (V_oligomycin_) to obtain the minimal respiration. Maximized respiration was monitored by adding the uncoupler FCCP (1 μM) (V_FCCP_). (B) V_basal_/V_oligomycin_ index was generated to determine the level of coupling between respiration and oxidative phosphorylation in SCR and *VCP* KD cells. (C) V_basal_/V_FCCP_ index was generated to verify the function of ETC complexes of SCR and *VCP* KD SH-SY5Y cells. (D and E) Stable SCR and *VCP* KD cells (clones V#1, V#2, V#3, V#4, and Pool) were permeabilized and oxygen consumption was measured in state 3 (V_3_) (in which 50 nmol ADP were provided) and state 4 (V_4_) (in which no ADP is present). (D) The respiratory control ratio (RCR), a ratio of V_3_ to V_4_, was then generated. (E) The ADP consumed per atom of oxygen (ADP/O) was also determined. The experiment was performed in the presence of substrates for complex I (5 mM of glutamate and malate). Data are represented as mean ± SEM from at least three independent experiments (n/s = nonsignificant differences; ^∗^p < 0.05; ^∗∗^p < 0.01 compared with values from SCR cells). See also Tables S2 and S3.

Both the decrease in NADH autofluorescence and the loss of ΔΨ_m_ observed in the VCP-deficient cells could reflect either an increased mitochondrial proton leak and uncoupling, in which case oxygen consumption would be increased in these cells, or a lack of substrates, in which case it would be decreased. The rate of oxygen consumption was thus measured using a Clark oxygen electrode in three clonal populations of stable *VCP* KD SH-SY5Y cells. VCP protein expression levels were reduced by approximately 90% in stable *VCP* KD SH-SY5Y cells compared to stable SCR SH-SY5Y cells (Figure S1B). The basal rate of oxygen consumption was significantly increased in VCP KD cells compared to control (Figure 3A; for numbers see Table S2). Furthermore, addition of oligomycin resulted in an inhibition of oxygen consumption in control cells but not in *VCP* KD cells, while the uncoupler FCCP increased oxygen consumption to the same maximal values in both cell populations. Calculation of V_basal_/V_oligomycin_ and V_basal_/V_FCCP_ revealed a decrease in the V_basal_/V_oligomycin_ index, suggesting an increase in mitochondrial respiration (Figure 3B; for numbers see Table S2). No differences in the V_basal_/V_FCCP_ index were observed between *VCP* KD and SCR cells, showing that ETC complexes are not damaged and they are working at a similar rate under normal conditions (Figure 3C; for numbers see Table S2).

To further analyze ETC and OXPHOS functionality and coupling, we permeabilized stable *VCP* KD and SCR cell lines using a low concentration of digitonin (40 μM) and basal oxygen consumption rates were measured in the presence of external substrates for the ETC in the absence of ADP. We then added 50 nmol ADP to establish state 3 (V3) respiration. Upon consumption of this ADP, mitochondria resumed an inhibited state, termed state 4 (V4). Respiratory control ratio (RCR) is the ratio of V3 to V4 and is considered an indicator of coupling of OXPHOS and respiration. RCR values confirmed the uncoupling of mitochondrial respiratory chain from oxidative phosphorylation in stable *VCP* KD cells compared to control (Figure 3D; for numbers see Table S3). The “ADP/O” ratio, expressed as the oxygen consumed per nmol ADP added during V3, indicates the efficiency of oxidative phosphorylation. ADP/O ratios indicated that oxidative phosphorylation efficiency was reduced by more than 30% in *VCP* KD cells compared to SCR cells (Figure 3E; for numbers see Table S3). Taken together, these data show that the respiratory rate, driven by the loss of potential and oxidation of the NADH pool, is increased in VCP-deficient cells and that VCP deficiency increases the mitochondrial proton leak causing uncoupling between respiration and OXPHOS.

Mitochondrial uncoupling may occur through a variety of mechanisms including altered lipid peroxidation. Using the fluorescent ratiometric oxidation-sensitive dye C11 BODIPY581/591, we therefore determined the levels of lipid peroxidation in VCP-deficient cells. The assay is based on the observation that oxidation of the dye by peroxyl radicals causes a shift in fluorescence emission from red to green. Figure S3A shows the green/red fluorescence ratios over time in a single experiment, while Figure S4B shows the average rate of increase in green/red fluorescence over three independent experiments. Fibroblasts carrying *VCP* mutations exhibit similar lipid peroxidation rates when compared to controls (Figures S3A–S3C). These results suggest that uncoupling in these cells is not related to changes or alterations in lipid peroxidation rates.

### ATP Levels Are Decreased in VCP-Deficient Cells

**Figure 4:**
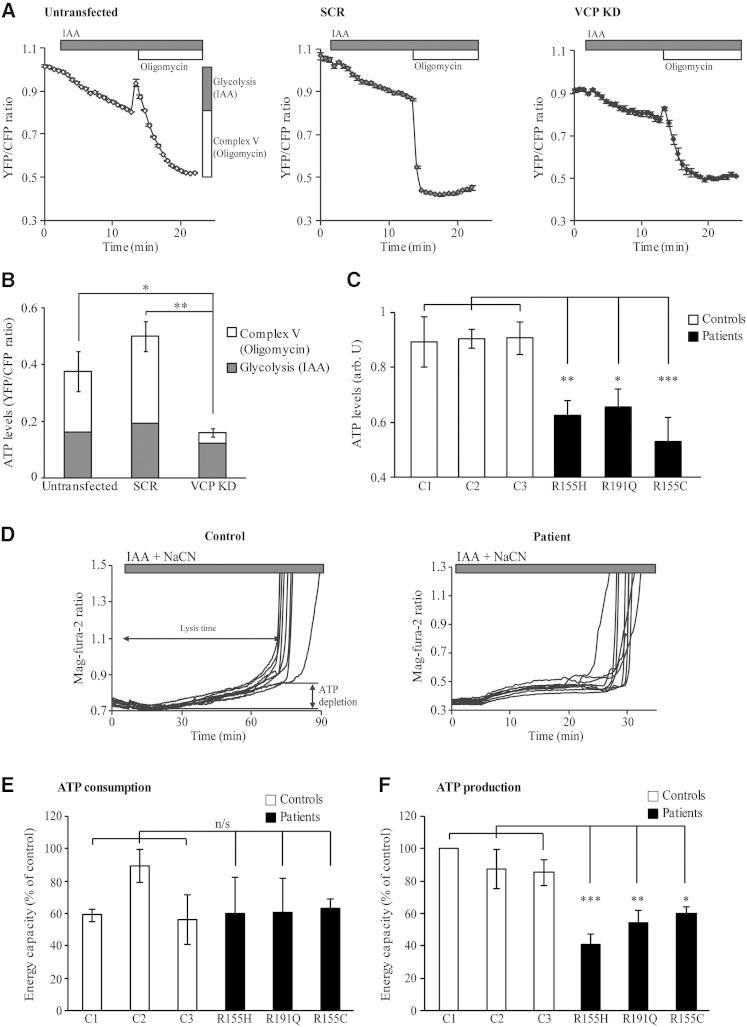
Lower ATP Production Reflects Lower ATP Levels in VCP-Deficient Cells (A–C) ATP levels in untransfected, SCR, and *VCP* KD SH-SY5Y cells were measured using a FRET-based method by transfection of the ATP indicator AT1.03. Cells were subjected to imaging and ratio-metric analysis of the yellow- (YFP) and cyan- (CFP) fluorescent proteins, allowing estimation of ATP kinetics within single cells by confocal microscopy. ATP levels from glycolysis or from oxidative phosphorylation are described graphically (A, left). (A) Representative experiments of time-dependent YFP/CFP ratios from untransfected, SCR, and *VCP* KD cells (mean ± SEM of at least 10 cells on a single coverslip). The initial YFP/CFP ratio was obtained when no ATP synthesis was inhibited, once glycolysis was inhibited with iodoacetic acid (IAA, 100 μM) and after inhibition of ATPase with oligomycin (0.2 μg/ml) (A, left). (B) Quantified ATP levels from glycolysis (dark gray bars) or from oxidative phosphorylation (white bars). (C) ATP levels in fibroblasts were measured using a bioluminescent assay based on the luciferin-luciferase system. (D) ATP consumption/production levels were assessed by using the Mg^2+^-sensitive fluorescent probe Mag-Fura. ATP depletion and time of cell lysis are described graphically (D, left). Representative traces from control (left) and patient fibroblasts (right) after inhibition of respiration and glycolysis. (E) Energy capacity as seen as time to cell lysis in response to inhibition of ATP synthesis (both glycolysis with IAA and oxidative phosphorylation with oligomycin) compared to control values expressed as percentages. The panel shows the ATP consumption in fibroblasts. (F) Energy capacity as seen as time to cell lysis in response to NaCN (1 mM) and IAA (0.2 μM) compared to control values expressed as percentage. The panel shows the ATP production in fibroblasts. In all cases, the error bars represent the mean ± SEM of at least three independent experiments carried out in triplicate (n/s = nonsignificant differences; ^∗^p < 0.05; ^∗∗^p < 0.01; ^∗∗∗^p < 0.005 compared with control values). See also Figure S4.

Basal cellular ATP levels are determined by the rates of ATP production (oxidative phosphorylation and glycolysis) and consumption. To monitor ATP levels in live VCP-deficient cells, we used a FRET-based ATP sensor. In a first subset of experiments, control and *VCP* KD SH-SY5Y cells were treated with 100 μM glycolytic inhibitor iodoacetic acid (IAA) to monitor the ATP levels generated from glycolysis and then with 0.2 μg/ml oligomycin to determine the ATP levels generated by the ATP synthase (Figures 4A, 4B, and S4A). In a second group of measurements, ATP synthase was inhibited prior to inhibition of glycolysis (Figure S4B). In all experiments, basal ATP levels were measured prior to treatment with inhibitors. Figure 4A shows traces from a representative experiment in which ATP levels were measured in untransfected, SCR, and *VCP* KD cells. The relative ATP levels generated by glycolysis and the ATP synthase as seen as a reduction in the YFP/CFP ratio after addition of inhibitors are represented in Figure 4B. A not statistically significant decrease in ATP levels was observed in *VCP* KD cells after inhibition of glycolysis by IAA (Figures 4B and S4A). However, ATP levels were significantly lower in *VCP* KD cells compared to controls after inhibition of ATP synthase by oligomycin (Figure 4B and S4A) (YFP/CFP: untransfected = 0.21 ± 0.07, n = 3; SCR = 0.30 ± 0.05, n = 4; *VCP* KD = 0.04 ± 0.01, n = 4). Interestingly, when glycolysis was inhibited after ATP synthase, control and *VCP* KD SH-SY5Y cells showed no decrease in ATP levels in response to IAA (Figure S4B). Due to the low efficiency of transfection in primary patient fibroblasts, a bioluminescent assay based on the luciferin-luciferase system was used to detect the ATP levels in these cells. In all three patient fibroblast lines, ATP levels were significantly decreased compared to age-matched control fibroblasts (luminescence arbitrary units: patient 1 = 0.63 ± 0.05, n = 7; patient 2 = 0.66 ± 0.07, n = 5; patient 3 = 0.53 ± 0.09, n = 7; control 1 = 0.90 ± 0.09, n = 7; control 2 = 0.91 ± 0.03, n = 6; control 3 = 0.90 ± 0.06, n = 4) (Figure 4C). These experiments show that *VCP* pathogenic mutations also lead to decreased ATP levels. The energy capacity was then measured in VCP-deficient fibroblasts to determine the cause of low ATP levels in these cells. The energy capacity of a cell is defined as the time between application of inhibitors of glycolysis/ATP-synthase (i.e., cessation of ATP production) and the time of cell lysis (i.e., energetic collapse due to total ATP depletion and inability to maintain Ca^2+^ homeostasis) (Yao et al., 2011) (Figure 4D). The ATP consumption rates were estimated by measurement of the energy capacity after inhibition of glycolysis and F_1_F_0_-ATP synthase with IAA (100 μM) and oligomycin (0.2 μg/ml), respectively, showing no differences between patient and control fibroblasts (Figure 4E). However, the ATP production rates in patient fibroblasts monitored by inhibition of glycolysis (IAA, 100 μM) and respiration (NaCN, 1 mM) were found to be significantly decreased compared to controls (Figure 4F) (energy capacity: patient 1 = 41% ± 6%; patient 2 = 55% ± 8%; patient 3 = 60% ± 6%; control 1 = 100% ± 0%; control 2 = 88% ± 12%; control 3 = 86% ± 8%; n = 3). These results show that VCP-deficient cells generate less ATP than control cells but also demonstrate the vulnerability of these cells to chemical ischemia (Figure 4F).

## Discussion

As the energy factories of the cells, mitochondria play a vital role in neurons, in which oxidative phosphorylation is the main source of ATP. Previous studies have shown that pathogenic *VCP* mutations modulate VCP ATPase activity in vitro (Halawani et al., 2009) and that they are associated with altered cellular ATP levels in *Drosophila* (Chan et al., 2012; Chang et al., 2011; Manno et al., 2010). In this study, we investigated the mitochondrial bioenergetics in VCP-deficient cells and in fibroblasts with *VCP* mutations from IBMPFD patients. We show that loss of VCP function is associated with decreased ΔΨ_m_ in the above cell models and in mouse cortical primary neurons and astrocytes. VCP deficiency further results in increased mitochondrial respiration and uncoupling. These observations are accompanied by decreased ATP levels due to lower ATP production.

A number of prior studies have observed altered mitochondrial respiratory complex function in ALS disease models including postmortem brain and spinal cord tissue (Bowling et al., 1993; Wiedemann et al., 2002), patient lymphocytes (Ghiasi et al., 2012), and a transgenic mouse model of ALS (Jung et al., 2002). Despite these findings, there remains some controversy surrounding the dysfunction of mitochondrial respiratory chain complexes in ALS, and we previously found normal activity in muscle, myoblasts, fibroblasts, and cybrids from patients (Bradley et al., 2009). Accordingly, our results strongly suggest that there is no impairment of mitochondrial respiratory complexes in any of the fibroblasts from the IBMFPD patients carrying the *VCP* pathogenic mutations.

We observed that ΔΨ_m_ was decreased in all the VCP-deficient cell models. ΔΨ_m_ is a key indicator of mitochondrial viability, as it reflects the pumping of hydrogen ions across the inner membrane during the process of electron transport, the driving force behind ATP production. We showed that VCP-deficient cells are still able to maintain their ΔΨ_m_ through respiration, since addition of oligomycin, the inhibitor of the F_1_F_0_-ATP synthase, induced no response in *VCP* KD cells or a slight depolarization in patient fibroblasts. These data suggest that while ΔΨ_m_ is largely maintained by respiration in both SH-SY5Y cells and patient fibroblasts, in patient fibroblasts carrying *VCP* mutations, ATPase may be required to work in its reverse mode hydrolysing ATP in order to maintain the ΔΨ_m_.

In combination with low ΔΨ_m_, we observed decreased NADH redox index in our VCP-deficient cell models compared to control cells, indicating increased respiration. These results are consistent with the higher oxygen consumption rates and lower RCR values obtained in VCP-deficient cells, indicating mitochondrial uncoupling between respiration and oxidative phosphorylation. In agreement with these observations, ADP/O values were lower in VCP-deficient cells, confirming that the oxidative phosphorylation efficiency is decreased in these cells. In order to compensate for the accumulation of uncoupled mitochondria, cells may stimulate mitochondrial biogenesis (for review, see Perez-Pinzon et al., 2012). However, no difference in mitochondrial mass was observed in our VCP-deficient neuroblastoma cells compared to controls (Figure S3), suggesting that the uncoupling observed in these cells is due to physiological rather than structural mitochondrial abnormalities.

Mitochondrial uncoupling has previously been observed in different models of neurodegeneration (Papkovskaia et al., 2012; Plun-Favreau et al., 2012; White et al., 2011). It may be induced via various mechanisms including altered mitochondrial membrane integrity due to excessive lipid peroxidation-derived free radical production (Brookes et al., 1998; Chen and Yu, 1994). Here we show that lipid peroxidation levels are not altered in VCP-deficient cells, indicating that uncoupling is unlikely to occur through this mechanism in these cells. Alternative possibilities include that VCP deficiency is associated with a deregulation of the uncoupling proteins (UCPs) or the adenine nucleotide translocase (ANT1), both of which play an important role in regulating the coupling of mitochondrial respiratory chain to oxidative phosphorylation. Interestingly, the levels of UCPs, key regulators of mitochondrial function, have previously been shown to be altered in skeletal muscle biopsies from rat and mouse ALS models and human ALS patients (Dupuis et al., 2003; Patel et al., 2010; Smittkamp et al., 2010). Further experimental investigation is required to determine whether altered levels or function of UCPs are observed in VCP-deficient cells. UCPs could dissipate the proton gradient, generated in the intermembrane space by the increased respiration observed in VCP-deficient cells, into heat. ANT1 was also found to be highly expressed in a transgenic mouse model of ALS (Martin et al., 2009). This ADP/ATP translocase is responsible for the transport of adenine nucleotides across the mitochondrial inner membrane, but it is also thought to have an intrinsic uncoupling property (Brand et al., 2005). Under healthy conditions, the ΔΨ_m_ generated by respiration is used by ANT1 for translocation of cytosolic ADP to the mitochondrial matrix and further generation of ATP. A slight increase in oxygen consumption when ADP was added in permeabilized cells points to a possible deregulation in the ADP/ATP translocase induced by the VCP deficiency. Further physiological and biochemical experiments will be necessary to determine the possible roles of UCPs and ANT1 in the VCP-deficient cells.

VCP has been proposed to participate in the clearance of depolarized mitochondria through selective autophagy (Tanaka et al., 2010), raising the possibility that loss of *VCP* mutations allows the accumulation of damaged, uncoupled mitochondria that would usually be degraded. However, genetic mutations in other members of this pathway (*PINK1* and *Parkin*) lead to a markedly different phenotype in patients (Vincent et al., 2012; Yao et al., 2011) and mitochondrial uncoupling has not been reported in cells lacking either protein, suggesting a more direct role for VCP in the mitochondrial uncoupling we observe.

A number of studies in different ALS models have linked mitochondrial deficiency and altered ATP levels to the pathogenesis of the disease (Ghiasi et al., 2012; Mattiazzi et al., 2002). Browne and colleagues have suggested that the decreased ATP levels they observed in ALS transgenic mice could be due to uncoupling (Browne et al., 2006) and IBMPFD-causing *VCP* mutations were associated with altered ATP levels in *Drosophila* (Chang et al., 2011). Here we further confirm that *VCP* mutations lead to reduced ATP levels in patient fibroblasts carrying three independent pathogenic mutations, and we show that this is the result of lower ATP production rather than higher ATP consumption. In VCP-deficient flies, on the other hand, altered ATP levels were suggested to be the result of higher rates of ATP consumption (Chan et al., 2012; Chang et al., 2011). These discrepancies could reflect the different metabolism between flies and mammals and may be further explained by the use of different research methods. ATP depletion was previously shown to induce cytotoxicity, as the cells are no longer able to maintain their ionic homeostasis and flood with calcium (reviewed by Abramov and Duchen, 2010; Bolaños et al., 2009). Our data confirm that VCP-deficient fibroblasts are more vulnerable to cytotoxicity than control cells after depletion of ATP.

Together, our results suggest that the pathogenic *VCP* mutations have a dominant-negative effect that presumably arises from a loss of function of the hexameric protein through poisoning by the mutant subunits. The ΔΨ_m_ measurement carried out in SH-SY5Y cells overexpressing *VCP* WT or pathogenic mutants as well as the rescue experiment in the stable *VCP* KD SH-SY5Y cells (Figures 1E and 1F) strengthen this hypothesis. This inhibitory effect of the mutant subunits on the wild-type subunits has previously been shown for Torsin A, another member of the AAA ATP-ase family and its torsin dystonia-associated mutants (Goodchild et al., 2005). Two of the *VCP* mutations harbored by the patient fibroblasts are located at the N domain of the protein (R155H and R155C), whereas the third one is located in the link between the N and the D1 domain (R191Q). It was previously shown that none of IBMPFD mutations in *VCP* disrupt gross hexamer formation (Halawani et al., 2009).

The mitochondrial dysfunction observed in this study may help to explain the myopathic phenotype of VCP patients. Dementia is unusual in mitochondrial disorders, but mitochondrial dysfunction has also been suggested to be important in a number of central degenerative disorders such as SOD1-associated ALS (for review, see Dupuis et al., 2003).

In summary, we have characterized the mitochondrial pathology in fibroblasts from IBMFPD patients carrying *VCP* mutations and identified a possible mechanism of neurotoxicity. Our data suggest that VCP deficiency leads to severe mitochondrial uncoupling, resulting in decreased ATP production and subsequent depletion of cellular ATP. This lack of ATP in turn renders VCP-deficient cells significantly more vulnerable to cytotoxicity in response to any further inhibition of mitochondrial respiration, and we propose that it is this vulnerability to ischemic conditions that may ultimately cause the neuronal death observed in the IBMFPD patients.

## Experimental Procedures

Detailed Experimental Procedures can be found in the Supplemental Experimental Procedures. All samples were collected with the written consent of participants and formal ethical approval from the National Hospital for Neurology and Neurosurgery–Institute of Neurology Joint Research Ethics Committee (London, UK).

## Supplemental Information

Document S1. Figures S1–S4, Tables S1–S3, and Supplemental Experimental Procedures
