# Supplementary material for: Pathogenic VCP Mutations Induce Mitochondrial Uncoupling and Reduced ATP Levels
Source: Neuron. 2013 Apr 10;78(1):57–64. doi: 10.1016/j.neuron.2013.02.028 (PMC3843114; doi:10.1016/j.neuron.2013.02.028)
Supplement: Document S1. Figures S1–S4, Tables S1–S3, and Supplemental Experimental Procedures [file mmc1.pdf]

Neuron, Volume *78*

Supplemental Information

Pathogenic *VCP* Mutations

Induce Mitochondrial Uncoupling

and Reduced ATP Levels

Fernando Bartolome, Hsiu-Chuan Wu, Victoria S. Burchell, Elisavet Preza, Selina Wray, Colin J. Mahoney, Nick C. Fox, Andrea Calvo, Antonio Canosa, Cristina Moglia, Jessica Mandrioli, Adriano Chiò, Richard W. Orrell, Henry Houlden, John Hardy, Andrey Y. Abramov, and Helene Plun-Favreau

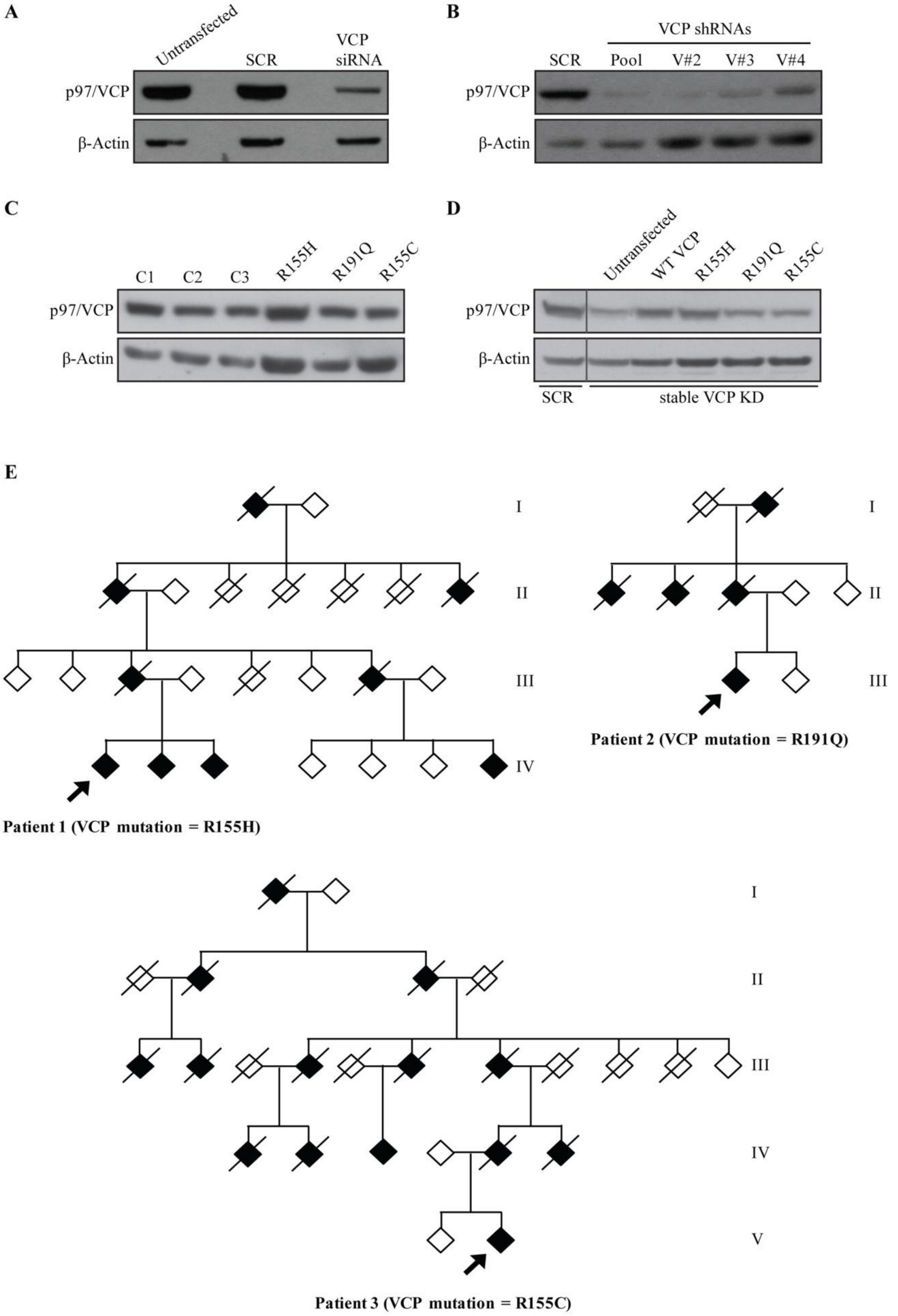
A
Untransfected
SCR
VCP siRNA
p97/VCP
β-Actin
B
VCP shRNAs
SCR
Pool
V#2
V#3
V#4
p97/VCP
β-Actin
C
C1
C2
C3
R155H
R191Q
R155C
p97/VCP
β-Actin
D
Untransfected
WT VCP
R155H
R191Q
R155C
p97/VCP
β-Actin
SCR
stable VCP KD
E
I
II
III
IV
V
Patient 1 (VCP mutation = R155H)
Patient 2 (VCP mutation = R191Q)
Patient 3 (VCP mutation = R155C)

Figure S1. VCP protein levels and patient information. (**A**) Immunoblotting of whole cell lysates from untransfected and transiently transfected SH-SY5Y cells with SCR or VCP siRNA. (**B**) Immunoblotting of stable SCR and VCP knockdown cell lysates (clones V#2, V#3, V#4 and pool of all the shRNAs). (**C**) Immunoblotting showing the VCP levels of fibroblasts from patients carrying VCP mutations and aged-matcehd controls. (**D**) Immunoblotting showing the basal VCP levels in the stable SCR population and one of the stable VCP KD clones. The VCP levels were rescued after overexpression of the WT VCP and the R155H, R191Q and R155C mutations. In all cases β-Actin was used as loading control. (**E**) Family trees from *VCP* mutations carriers. Affected and non-affected members from the families are labelled in black and white, respectively. Black arrows indicate the selected patients within each family. Related to **Figure 1**.

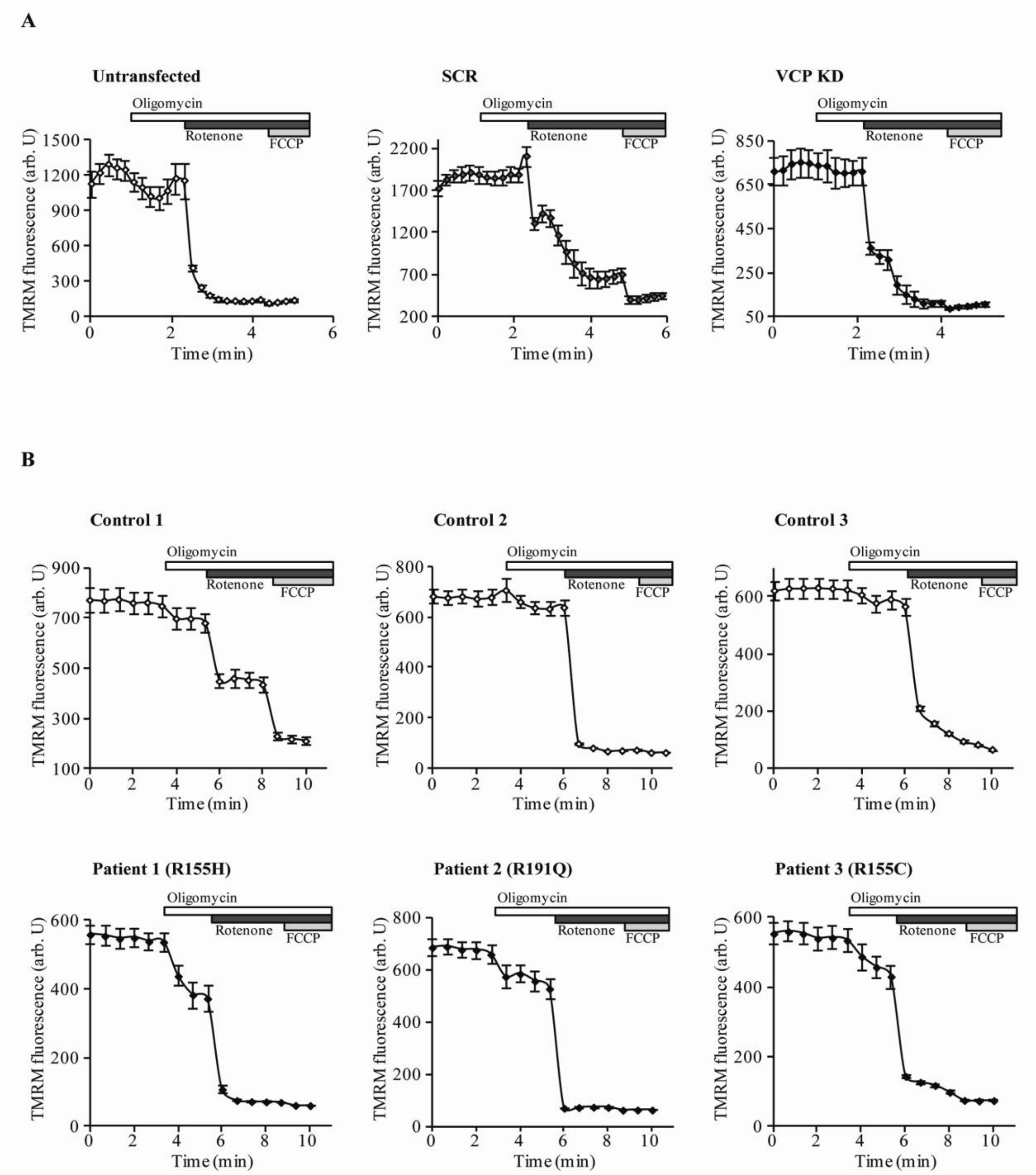

Figure S2. $\Delta\Psi_m$ in VCP deficient cells is maintained by respiration. Representative TMRM traces from untransfected, SCR and VCP KD SH-SY5Y cells (**A**) and three control fibroblasts (**B, upper panels**) and fibroblasts from patients carrying the VCP pathogenic mutations R155H, R191Q and R155C (**B, bottom panels**), showing responses to oligomycin (0.2 μM), rotenone (5 μM) and FCCP (1 μM). Data represent the mean ± SEM of at least 20 cells on a single coverslip. Related to **Figure 1**.

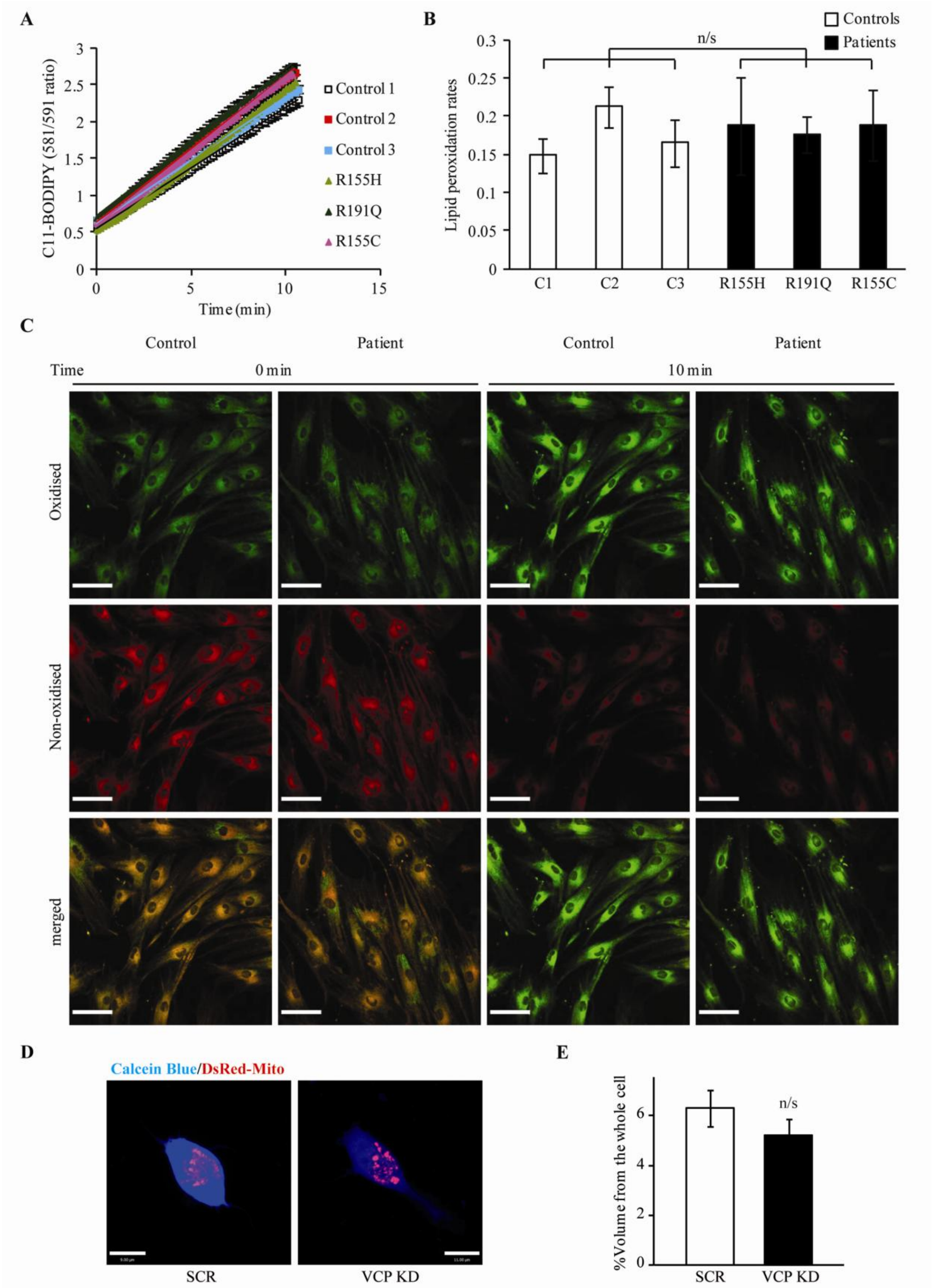

Figure S3. Ucoupling in VCP deficient cells is not due to altered rates of lipid peroxidation. Mitochondrial mass is not affected by VCP deficiency. (A–C) Lipid

peroxidation rates were measured by live cell imaging in control and patient fibroblasts using the fluorescent ratiometric oxidation-sensitive dye C11 BODIPY581/591 (C11-BO). C11-BO shifts its fluorescence from red to green in a time-dependent manner and these changes can be detected by confocal microscopy. (**A**) Time-course representative traces of the green/red fluorescence ratios from control and patient fibroblasts are shown. (**B**) Quantified lipid peroxidation rates from control and patient fibroblasts. (**C**) Confocal-representative images of one control and one patient fibroblasts (all controls and all patients showed similar confocal images) showing the green (oxidised dye), red (non-oxidised dye), and merged fluorescence images at initial time of recording (0 min) and after 10 minutes (10 min) of C11-BO treatment. Scale bar is 42 μm. n/s indicates non-significant differences. (**D**) Representative images displaying stable SCR and VCP KD SH-SY5Y cells loaded with Calcein Blue (cell cytosol in blue) and expressing the indicated DsRed-Mito construct (red) showing the mitochondria. (**E**) Mitochondrial mass appears with no changes in VCP deficient cells compared with SCR control. In all cases the fluorescence was measured in at least 15 cells on a single coverslip and the bars represent the mean ± SEM of at least 3 independent experiments (n/s = non-significant differences).

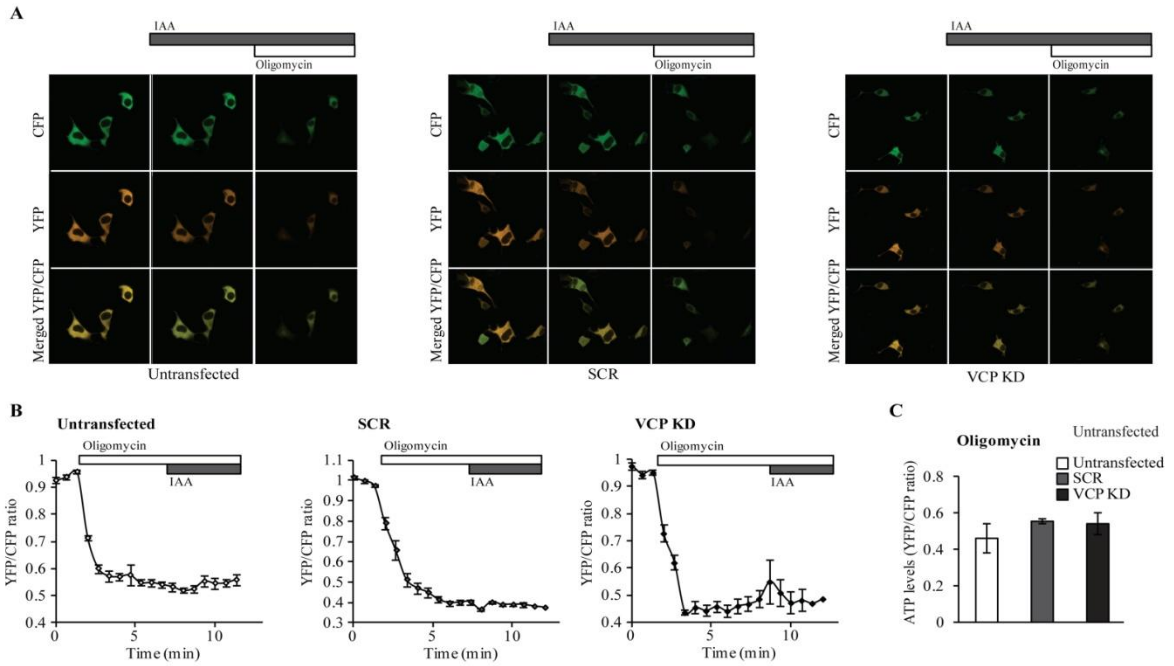

Figure S4. ATP levels inside living cells. (**A**) Representative images from the yellow - (YFP) and cyan- (CFP) fluorescent proteins and merged images after addition of IAA (100 μM) and oligomycin (0.2 μg/ml) in untransfected, SCR and VCP KD SH-SY5Y cells. Representative traces and the corresponding histograms quantifying this experiment can be seen in Figures 4A and B. (**B**) Kinetics of ATP levels measured by inhibition of ATP synthase followed by inhibition of glycolysis. Representative experiments of time-dependent YFP/CFP ratios from untransfected, scrambled and VCP KD SH-SY5Y cells. Data represent the mean ± SEM of at least 10 cells on a single coverslip. (**C**) ATP levels from oxidative phosphorylation measured by inhibiting ATP synthase and then glycolysis obtained by the corresponding YFP/CFP ratios. The bars represent the mean ± SEM of at least 3 independent experiments. n/s indicates non-significant differences. Related to **Figure 4**.

Table S1. Donor's information

	Sex	Current Age	Age of onset	Family history	Clinical features	Clinical diagnosis	VCP mutation
**Control 1**	M	57	-	-	Normal	Normal	No
**Control 2**	M	66	-	-	Normal	Normal	No
**Control 3**	F	50	-	-	Normal	Normal	No
**Patient 1**	M	53	40	AD	Presented with lower limb pain and weakness. Increasing fatigue and dyspnoea. Examination revealed predominantly proximal limb wasting and weakness, areflexia and episodic memory problems with increased irritability. Executive functioning was abnormal as was recognition memory for faces.	IBMPFD	R155H
**Patient 2**	M	45	36	AD	First symptom of finger weakness, then progressive distal and proximal limb weakness and wasting, wheelchair in the early 40's then speech problems. Later into the illness memory and emotional problems have occurred.	IBMPFD	R191Q
**Patient 3**	F	43	35	AD	Presented with difficulty cloths over her head and muscle ache after swimming. Progressive problems with weakness and wasting proximally and later distally in the lower then the upper limbs, fatigue, muscle pain, cramps, weight loss and later frontal executive signs.	IBMPFD	R155C

Table S2. Mitochondrial bioenergetics I

	SCR	V#2	V#3	Pool
**Basal**	1.02 ± 5.01 x 10^{-6}	2.08 ± 0.36 *	2.26 ± 0.36 *	2.30 ± 0.25 *
**Oligomycin**	0.60 ± 0.03 *	1.71 ± 0.37	1.59 ± 0.23	1.80 ± 0.30
**FCCP**	1.33 ± 0.11 *	2.46 ± 0.58	2.74 ± 0.54	2.78 ± 0.39
$\mathbf{V_{basal}/V_{oligomycin}}$	1.75 ± 0.06	1.25 ± 0.07 *	1.32 ± 0.12 *	1.20 ± 0.18 *
$\mathbf{V_{basal}/V_{FCCP}}$	0.82 ± 0.03	0.92 ± 0.10	0.89 ± 0.06	0.85 ± 0.10

Basal, oligomycin and FCCP corresponds to the rates of oxygen consumption (nmol O2/min/10^6 cells) in basal condition, in the presence of the ATPase inhibitor oligomycin (0.2 µg/ml) and in the presence of the uncoupler FCCP (1 µM), respectively. Data are represented as mean ± SEM from at least 4 independent experiments (* = P<0.05. Related to **Figure 3**.

Table S3. Mitochondrial bioenergetics II

	SCR	V#1	V#2	V#3	V#4	Pool
**RCR**	1.65 ± 0.15	1.13 ± 0.02*	1.08 ± 0.03*	1.26 ± 0.12*	1.12 ± 0.04*	1.22 ± 0.06*
**ADP/O**	0.99 ± 0.01	0.26 ± 0.03**	0.50 ± 0.05**	0.70 ± 0.02**	0.25 ± 0.03**	0.74 ± 0.10**

RCR (respiratory control ratio); ADP/O (ADP consumed per atom of oxygen). The experiment was performed in the presence of substrates for complex I (5 mM of glutamate and malate). Data are represented as mean ± SEM from at least 3 independent experiments (* = P<0.05; ** = P<0.01 compared with values from SCR cells). Related to **Figure 3**.

SUPPLEMENTAL EXPERIMENTAL PROCEDURES

Donors

Fibroblasts were generated from a 4 mm skin punch biopsy taken under local anesthetic following informed consent. Biopsies were dissected into ~1 mm pieces and cultured in 5 cm^2 petri dishes in DMEM, 10% FBS, 1% L-Glutamine until fibroblasts were seen to grow out from the explants. When fibroblasts reached confluency, they were detached from culture dishes using TrypleE (Invitrogen) and transferred to larger culture vessels for further expansion and cryopreservation. Age, sex, age of onset, family history, clinical features, clinical diagnosis, and *VCP* mutations carried by the donors are given in Table S1. Family trees from patient 1, patient 2 and patient 3 are provided in Figure S1.

Mouse primary cortical cultures

Animal husbandry and experimental procedures were performed in full compliance with the United Kingdom Animal (Scientific Procedures) Act of 1986. For primary mouse cortical cultures, 6 C57-BL6 wt pups were culled between postnatal days P1–3. Cerebral hemispheres were pooled and removed into sterile 15 ml tubes containing 2 ml chilled HEPES-buffered salt solution (0.137 M NaCl, 5.4 mM KCl, 0.25 mM Na2HPO4, 0.44 mM KH2PO4, 1.3 mM CaCl2, 1.0 mM MgSO4, 4.2 mM NaHCO3, pH 7.4). The HEPES-buffered saline solution (HBSS) was then removed and replaced with 2 ml pre-warmed trypsin–EDTA solution for 5 min at room temperature. The cultures were pelleted by centrifugation at 2000 r.p.m. for 5 min, then the trypsin was gently aspirated and the cells washed first in pre-warmed HBSS, then in warm neurobasal medium containing 2% B27 supplement, 2 mM glutamine, 100 IU/ml penicillin and 100 IU/ml streptomycin. The cells were resuspended in 2 ml warm complete neurobasal medium and 3–4 drops of cells were plated per well on poly-L-lysine coated coverslips in 6-well plates. The cultures were incubated in a humidified incubator at

37°C with 5% CO_2 in air for 3–4 h, then 2 ml pre-warmed neurobasal medium added. Half the medium was replaced after 2 days, after which half the medium was replaced weekly. All live cell imaging experiments were performed between d10–d14 in culture.

Cell culture

Human neuroblastoma (SH-SY5Y) cells were purchased from the European Collection of Cell Cultures (Health Protection Agency, Salisbury, UK) and maintained as previously described (Muqit et al., 2006). Hek293T cells used for lentiviral particles generation were kindly provided by Dr. Klaus Wanish from the department of Clinical & Experimental Epilepsy, UCL-Institute of Neurology (London, UK). The subclonal SH-SY5Y cells were puromycin (1µg/ml) selected for stable knockdown of VCP (see below “stable VCP KD and SCR”). Unless otherwise stated, SH-SY5Y cells and fibroblasts were seeded at a density of 4 × 10^4 cells/cm^2, grown to 75–80% confluence and maintained at 37°C and 5% CO_2 in Dulbecco's modified Eagle's medium (DMEM) medium supplemented with 10% (v/v) foetal bovine serum (FBS), 2 mM L-glutamine and 1% (v/v) penicillin/streptomycin.

Antibodies

Anti-human VCP rabbit polyclonal antibody was obtained from Cell Signalling Technology (Beverley, MA, USA). Anti-human VCP mouse monoclonal antibody was purchased from Abcam (Cambridge, UK). Anti-human β-Actin rabbit polyclonal antibody was obtained from Sigma-Aldrich (Poole, UK).The anti-mouse and anti-rabbit secondary antibodies coupled to horseradish peroxidase and bovine immunoglobulins (IgG) were from Bio-Rad (Richmond, CA).

Plasmids and reagents

The non-targeting scramble siRNA and the targeted siRNA (siGenome SMARTpool) against human VCP and the Dharmafect transfection reagent were purchased from Dharmacon, Thermo Fisher Scientific (Waltham, MA, USA). The R155H, R191Q and R155C VCP vectors for the VCP mutants over-expression in the SH-SY5Y cells were created by site directed mutagenesis using the WT VCP EGFP vector from Addgene as template. The ATP-plasmid indicator encoding AT1.03 cDNA for ATP measurements was kindly provided for Hiromi Imamura from The Hakubi Center & Graduate School of Biostudies, (Kyoto University, Japan). The Effectene transfection reagent was from Qiagen (Hilden, Germany).

shRNA

Scramble and VCP KD shRNA SH-SY5Y cells were generated by lentiviral infection with either a pool of 5 shRNA vectors or individuals shRNA (Thermo Fischer) and subsequent clonal selection with antibiotic (puromycin 1μg/ml). Sequences from VCP shRNA were: clone 1 (V#1) = CCCTGTGGAGCACCCAGACAAA; clone 2 (V#2) = CCAGGGGGTTTCTGTTGCAAAA; clone 3 (V#3) = AAAGATGGATCTCATTGACCTA; clone 4 (V#4) = CCACAGTGTTGCTGAAAGGAAA; clone 5 (V#5) = AGCGCATTGTATCACAGTTGTT. Four different independent clones and pool with 90% approx reduction in gene expression were selected for use in all experiments. 1 clone expressing non targeting RNA vector was used as control (stable SCR). For the mouse cortical primary cultures SCR and VCP KD shRNA mouse specific (Thermo Fisher) were transfected with Effectene transfection reagent 48 hours before the experiments. The sequence from the mouse VCP shRNA was = ATCGCTGACAGAACGTCGG.

Measurement of mitochondrial membrane potential ($\Delta\Psi_m$)

For measurements of $\Delta\Psi_m$, SH-SY5Y cell cultures or fibroblasts plated on 25 mm coverslides were loaded with 40 nM tetramethyl rhodamine methyl ester (TMRM) in a HEPES-buffered salt solution (HBSS) (composed of 156mM NaCl, 3mM KCl, 2mM $MgSO_4$, 1.25mM KH_2PO_4, 2mM $CaCl_2$, 10mM glucose and 10mM HEPES; pH adjusted to 7.35 with NaOH) for 40 minutes at room temperature and keeping the dye present in the chamber at the time of recording. Confocal images were obtained using a Zeiss 710 VIS CLSM (Zeiss, Oberkochen, Germany) equipped with a META detection system and a × 40 oil immersion objective. TMRM was excited using the 560 nm laser line and fluorescence was measured above 580 nm. TMRM fluorescence intensity was quantified by removing all background signals and measuring the mean TMRM fluorescence intensity in the pixels containing mitochondria therefore, the signal is independent of mitochondrial mass. For basal $\Delta\Psi_m$ measurements, Z-stack images were obtained by confocal microscopy and analysed using Zeiss software (Zeiss). For analysis of response to mitochondrial toxins, images were recorded continuously from a single focal plane. TMRM is used in the redistribution mode to assess $\Delta\Psi_m$, and therefore a reduction in TMRM fluorescence represents mitochondrial depolarization.

Mitochondrial mass assessment

One day before the measurement, cells were transfected with DsRed-Mito vector (Addgene) which specifically targets mitochondria. 40 minutes before the experiment cells were loaded with Cell Trace Calcein Blue AM (Invitrogen). Transfected cells were easily identified by the presence of red mitochondrial fluorescence. High-resolution Z-stacks were acquired for ∼10 cells per group. The method enabled adequate visualization of the mitochondrial network. In order to quantify the changes in the mitochondrial network volume, the percentage co-

localization of the blue (cytosolic) signal and the red (mitochondrial) signal was attained. This ratio represents the volume of the cell that is occupied by mitochondria. The co-localization of these signals was set as 100% in vector-transfected cells, to enable a comparison between different cell groups. The histogram reflects the co-localization attained in independent experiments.

Measurement of NADH-FAD/redox indexes

NADH autofluorescence was measured using an epifluorescence inverted microscope equipped with a × 20 fluorite objective. Excitation light at a wavelength of 350nm was provided by a Xenon arc lamp, the beam passing through a monochromator (Cairn Research, Faversham, Kent, UK). Emitted fluorescence light was reflected through a 455 nm long-pass filter to a cooled CCD camera (Retiga, QImaging, Surrey, BC, Canada) and digitised to 12 bit resolution. Imaging data were collected and analysed using software from Andor (Belfast, UK). FAD autofluorescence was monitored using a Zeiss 710 VIS CLSM equipped with a META detection system and a × 40 oil immersion objective. Excitation was measured using the 454 nm Argon laser line and fluorescence was measured from 505 to 550 nm. Illumination intensity was kept to a minimum (at 0.1–0.2% of laser output) to avoid phototoxicity and the pinhole set to give an optical slice of ~2 μm.

Oxygen consumption measurement

To measure respiration rate in intact cells ∼5 × 10^7cells were re-suspended in respiration medium (HEPES-buffered salt solution) with 10 mM D-glucose in a Clark-type oxygen electrode thermostatically maintained at 37°C. The oxygen electrode was calibrated with air-saturated water, assuming 406 nmol O_2 atoms/ml at 37°C (Oxytherm system, Hansatech Instruments). The basal rate of oxygen consumption was measured. Then, 0.2 μg/ml oligomycin was added to monitor the minimal respiration rate coupled with oxidative

phosphorylation and finally, FCCP (0.5 μM) was added to establish maximal uncoupled respiratory rate.

The respiratory parameters were determined on isolated mitochondria by previous cell permeabilization with digitonin 40 μM and subsequent re-suspension in a hypotonic medium containing 135 mM KCl, 10 mM NaCl, 20 mM HEPES, 0.5 mM KH_2PO_4, 1 mM $MgCl_2$, 5 mM EGTA and 1.86 mM $CaCl_2$ at pH 7.1. Oxygen consumption was measured in the Clark-type oxygen electrode thermostatically maintained at 25 °C. Glutamate (5 mM) and malate (5 mM) were added to measure Complex I-linked respiration, succinate (5 mM) with rotenone (5 μM) were added to measure Complex II-linked respiration. The amount of ADP added to assess the state 3 of respiration was 50 nanomoles. All data were obtained using an Oxygraph Plus system with chart recording software.

Lipid peroxidation

C11-BODIPY581/591 (C11-BO) (Molecular Probes, Eugene, OR) is a sensitive fluorescent probe for indexing lipid peroxidation in model membrane systems and living cells undergoing a shift from red to green fluorescence emission upon oxidation (Drummen et al., 2002). C11-BODIPY 581/591 (1 μM) was loaded for 15 min at 20 °C. Coverslips were washed once and resuspended in 1 ml of HBSS. Ratiometric measurements of probe oxidation were taken over a 12 min time course. Fluorescence excitation was at 488 nm, green fluorescence was detected at 530 nm and red fluorescence at 670 nm.

ATP levels

In order to have the best reproducible results of the ATP levels inside cells we used different approaches depending on the cells. Control, SCR and transient VCP KD SH-SY5Y cells were transfected with the ATP-plasmid indicator encoding AT1.03 cDNA as previously was

described (Imamura et al., 2009). The ATP probe AT1.03 has been validated extensively for real-time monitoring of ATP levels in single living cells. AT1.03 measures only free ATP, not Mg^{2+}-bound ATP and thus fluorescence of the ATP indicator represents the free available ATP pool. One day after transfection, cells were subjected to imaging and ratio-metric analysis of the yellow- and cyan-fluorescent proteins allowed estimation of ATP kinetics within single cells. Cells were transfected by using Effectene transfection reagent (Qiagen) following the manufacturer instructions. Measurements of ATP levels with AT1.03 were performed on a confocal microscope (Zeiss 710 LSM) with an integrated META detection system. Images were obtained using a 63× oil-immersion objective to allow immediate measurement of the fluorescent signal over cells. Cyan fluorescent protein was excited with 405 nm, and emission from 460 to 510 nm was measured. The 405 nm laser line was used to excite yellow fluorescent protein, which was measured using a band-pass filter from 515 to 580 nm. Illumination intensity was kept to a minimum (at 0.1 – 0.2% of laser output) to avoid phototoxicity and the pinhole set to give an optical slice of ~2 μm. The measurements with the ATP probe were performed in HBSS medium plus Ca^{2+} and Mg^{2+} that has been used for all other experimental procedures.

ATP basal levels in fibroblasts were measured by using the Vialight plus assay kit (Lonza, Verviers, Belgium) following the manufacturer instructions. Basically, the assay is based upon the bioluminescent measurement of ATP that is present in all metabolically active cells. Fibroblasts were seeded the day before assay in a 24 well plate. 24 h later, cells were lysed and 100 μl of the lysates were transferred into a 96 well plate in triplicate. Then, 100 μl of ATP monitoring reagent plus was added to generate the luminescent signal. Two minutes later, luminescence was checked on a microplate reader using a FLUOstar omega plate reader (BMG Labtech Ltd) with a specialised luminescence head. The amount of ATP in fibroblasts

was referred to a standard curve with known concentrations of ATP and finally according to the amount of protein estimated by BCA method (Pierce).

Energy capacity

Measurement of cellular free magnesium using the Mg^{2+}-sensitive fluorescent probe Mag-Fura AM can be used as an indicator of ATP consumption/production as Mg^{2+} is released from Mg-ATP upon ATP hydrolysis (Leyssens et al., 1996). Blocking ATP production depletes ATP and releases Mg^{2+}, resulting in an increase in Mag-Fura fluorescence. When ATP levels are depleted to a critical threshold, ionic homeostasis cannot be maintained and the cell floods with calcium. In addition to its high affinity for Mg^{2+}, Mag-Fura is also a low-affinity Ca^{2+} indicator, so this energetic collapse can be observed as a sudden increase in Mag-Fura ratio. Thus the Mag-Fura dye allows the measurement of both the basal ATP level and the energy capacity of the cell. Mag-Fura AM dye was added with 2% pluronic acid for 30 min. The energy capacity of the cell was assessed as the time between application of inhibitors and the time of cell lysis. Fluorescence measurements were obtained using an Olympus epifluorescence inverted microscope with a ×20 fluorite objective. Excitation light from a xenon arc lamp was selected using a monochromator at 340 and 380 nm (Cairn Research, Faversham, UK). Emitted light passed through a long-pass filter to a cooled CCD camera (Retiga, QImaging, Canada) and digitized to a 12-bit resolution (Digital Pixel Ltd., UK). All imaging data were collected and analyzed using software from Andor (Belfast, UK). Cells were protected from phototoxicity by interposing a shutter in the light path to limit exposure between acquisitions of successive images.

Statistical Analysis

Data were generated from a minimum of three independent experiments, using at least 15 cells per experiment and replication in two to three different clones for stable VCP KD cells.

Statistical analysis and exponential curve fitting were performed using Origin 8.5 (Microcal Software Inc., Northampton, MA) software. Statistical significance for multiple comparisons was performed by one-way ANOVA followed by LSD correction. In some experiments it was necessary to normalize each experiment to controls in order to eliminate variability between experiments. In all cases, $P<0.05$ was considered significant (*$P<0.05$, **$P<0.01$, ***$P<0.001$). For all graphs, error bars represent mean ± SEM.

SUPPLEMENTAL REFERENCES

Drummen, G.P., van Liebergen, L.C., Op den Kamp, J.A., and Post, J.A. (2002). C11-BODIPY(581/591), an oxidation-sensitive fluorescent lipid peroxidation probe: (micro)spectroscopic characterization and validation of methodology. Free radical biology & medicine *33*, 473-490.

Imamura, H., Nhat, K.P., Togawa, H., Saito, K., Iino, R., Kato-Yamada, Y., Nagai, T., and Noji, H. (2009). Visualization of ATP levels inside single living cells with fluorescence resonance energy transfer-based genetically encoded indicators. Proceedings of the National Academy of Sciences of the United States of America *106*, 15651-15656.

Leyssens, A., Nowicky, A.V., Patterson, L., Crompton, M., and Duchen, M.R. (1996). The relationship between mitochondrial state, ATP hydrolysis, [Mg2+]i and [Ca2+]i studied in isolated rat cardiomyocytes. The Journal of physiology *496 (Pt 1)*, 111-128.

Muqit, M.M., Abou-Sleiman, P.M., Saurin, A.T., Harvey, K., Gandhi, S., Deas, E., Eaton, S., Payne Smith, M.D., Venner, K., Matilla, A., *et al.* (2006). Altered cleavage and localization of PINK1 to aggresomes in the presence of proteasomal stress. J Neurochem *98*, 156-169.
